# Drosophilidae feeding on animals and the inherent mystery of their parasitism

**DOI:** 10.1186/s13071-014-0516-4

**Published:** 2014-11-18

**Authors:** Jan Máca, Domenico Otranto

**Affiliations:** Czech Entomological Society, Praha, Czech Republic; Department of Veterinary Medicine, University of Bari, 70010 Valenzano Bari, Italy

**Keywords:** Drosophilidae, Steganinae, zoophagy, parasitism, lachryphagy, *Phortica variegata*, *Thelazia callipaeda*

## Abstract

Insect evolution, from a free to a parasitic lifestyle, took eons under the pressure of a plethora of ecological and environmental drivers in different habitats, resulting in varying degrees of interactions with their hosts. Most Drosophilidae are known to be adapted to feeding on substrates rich in bacteria, yeasts and other microfungi. Some of them, mainly those in the Steganinae subfamily, display a singular behaviour, feeding on animal tissues or secretions. This behaviour may represent an evolving tendency towards parasitism. Indeed, while the predatory attitude is typical for the larval stages of a great proportion of flies within this subfamily, adult males of the genera *Amiota*, *Apsiphortica* and *Phortica* display a clearly zoophilic attitude, feeding on the lachrymal secretions of living mammals (also referred as to lachryphagy). Ultimately, some of these lachryphagous species act as vectors and intermediate hosts for the spirurid nematode *Thelazia callipaeda*, which parasitizes the eyes of domestic and wild carnivores and also humans. Here we review the scientific information available and provide an opinion on the roots of their evolution towards the parasitic behaviour. The distribution of *T. callipaeda* and its host affiliation is also discussed and future trends in the study of the ecology of Steganinae are outlined.

**Electronic supplementary material:**

The online version of this article (doi:10.1186/s13071-014-0516-4) contains supplementary material, which is available to authorized users.

## Introduction

### Initial stages of parasitism in insects

Insects and arachnids of medical and veterinary concern (e.g., mosquitoes, sand flies, stable flies, black flies, and ticks) have been studied extensively over the centuries, primarily because of the effect of their parasitic feeding habits on many species of domestic and wild animals, and humans. Indeed, these arthropods affect the health, welfare and production of animals through the transmission of disease-causing pathogens or just through biting them, therefore causing blood loss, allergic reactions, and/or nuisance and disturbance [1]. Evolution of arthropods, from a free to a parasitic lifestyle, took eons under the pressure of a wide range of ecological and environmental drivers, resulting in varying degrees of interactions with their hosts, e.g. from virtually necrophagous larvae, occasionally also causing facultative myiasis, to obligate parasitism. However, scientific information on the insect taxa that evolved only partial parasitic interactions with their hosts, is scant [2,3], and it puts them in a group of organisms of an as yet undefined parasitic status. For example, most Drosophilidae are known to be adapted to feeding on substrates rich in bacteria, yeasts and other fungi (e.g., decaying or fermenting fruit) [3]. However, some of them display a different feeding behaviour as they may feed on animal tissues or secretions (hereinafter referred as to “zoophagy”), therefore being of medical and veterinary importance. This particular behaviour may represent an evolving step towards parasitism. Indeed, there is still paucity of information on the natural history of these drosophilids, and great part of knowledge available to date derives from incidental findings from studies from the 19^th^ century [3]. The still limited entomological data on these insects is partly due to the difficulties in breeding these species under laboratory conditions [4]. Here we review the scientific information available and provide an opinion about the main drivers, which might have affected some drosophilid genera of the subfamily Steganinae towards parasitic behaviour.

## Review

### Zoophagy in Drosophilidae

*Drosophila melanogaster* (Diptera, Drosophilidae), the “vinegar fly”, is the quintessential member of this large family of insects, having had an enormous impact on various fields of science over the last century. Indeed, studies using this insect as a laboratory model have enabled great achievements in the field of developmental sciences (e.g. genetics, heredity, evolution, biochemistry, molecular biology, and cell biology) [5] as well as in applied disciplines such as neuroscience [6], the study of intellectual disability [7], metabolic disorders (e.g., obesity) [8] and oncology [9]. The reasons for the success of these tiny flies in science are related to their tolerance to environmental conditions, ease of rearing, short reproduction times, large numbers of offspring and the occurrence of several types of hereditary variations within a small genome size (i.e., four pairs of chromosomes). With the exception of this iconic species, as many as ca. 4,200 known species are included in the Drosophilidae family [10,11], displaying wide variations in morphology, behaviour, biology and ecology [12].

Two subfamilies are ranked in this family, namely the Drosophilinae, which at present includes 3,182 described species, and the less well-represented Steganinae, with 1,008 species [10]. Morphological characteristics of these subfamilies have been described in [13,14]. In Drosophilinae, zoophagous and/or commensal behaviour is restricted to larvae of a few scattered clades, classified at present as parts of the genera *Drosophila* (mainly *simulivora* group species), *Zygothrica* (1 species), *Scaptomyza* (subgenus *Titanochaeta*) and *Lissocephala* (1 species); altogether *ca*. 20 species [12]. Most other Drosophilinae are generally micromycetophagous (Sacharomycetales are clearly preferred substrates), although mycetophagy, saprophagy and phytophagy are typical for some genera or species groups [3].

**Figure 1:**
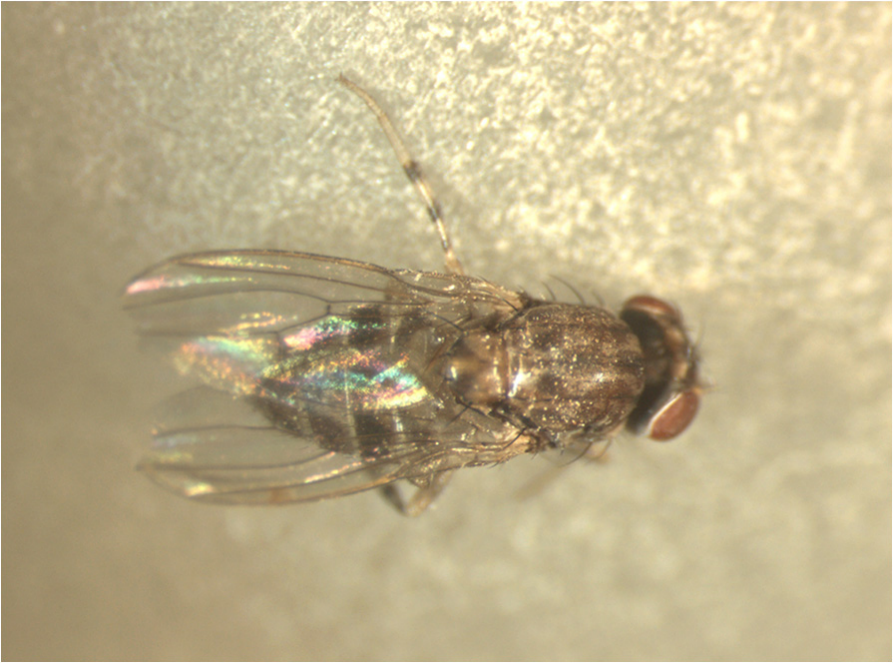
***Phortica variegata*** **.** Adult of *Phortica variegata* (Fallén), as representative of the subgenus *Phortica* s. str., containing more than 80 species known species with a similar appearance.

**Figure 2:**
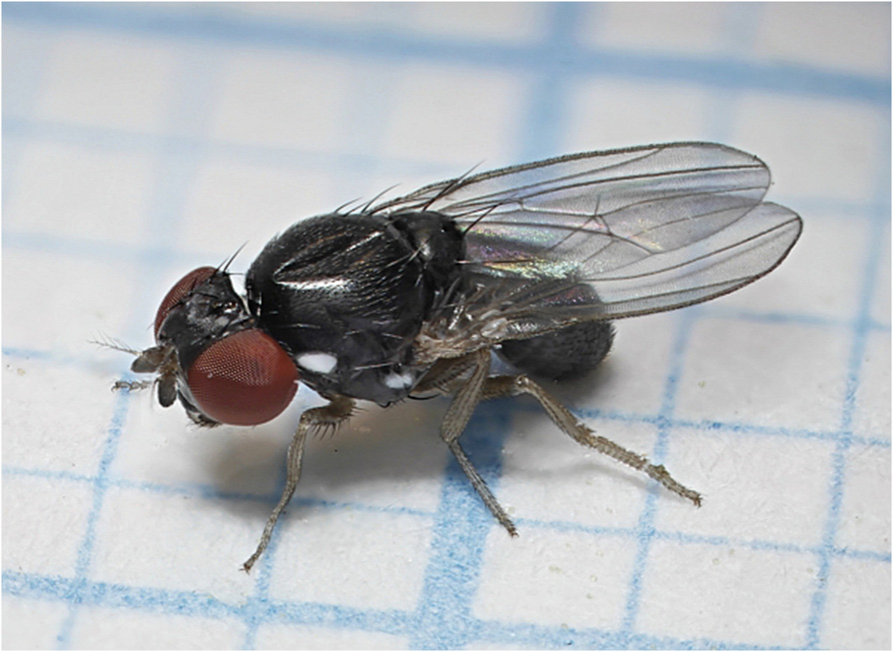
***Amiota filipes*** **.** Adult of *Amiota filipes* Máca. Most of the *Amiota* species are black or brown with silvery spots on face, postpronotum and katepisternum. Original, courtnesy K. Nielsen.

**Figure 3:**
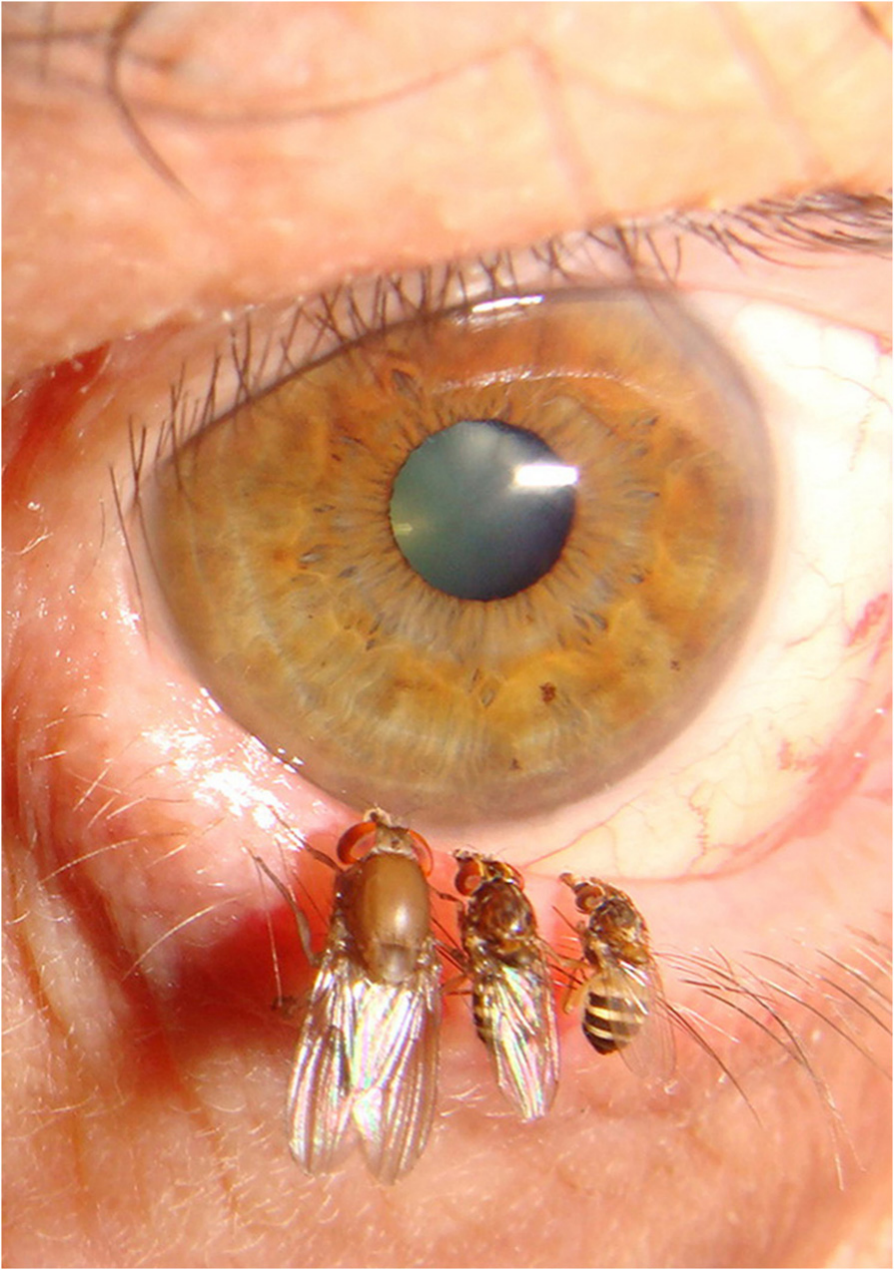
**Drosophilids at human eye in Thailand.** Three fruit flies (Drosophilidae) sipping tears from the human eye. Left: female *Apsiphortica longiciliata* Cao & Chen (females are very rarely lachryphagous); center: male *Phortica pseudotau* (Toda & Peng); right: male *Phortica* sp. (Photo H. Bänziger, from Bänziger et al., 2009 [2]).

As concerns the subfamily Steganinae, data based on cladistic and phylogenetic analyses showed that some of their morphological characters are related to numerous morphological convergences [13,14]. Predatory behaviour is typical for the larval stages of a great proportion of flies within this subfamily, although sometimes only in the initial stages (i.e., commensals that may ultimately kill their host). Members of the genera *Acletoxenus* and *Rhinoleucophenga*, as well as some species of *Cacoxenus* (subg. *Gitonides*) and *Leucophenga*, feed on Homoptera. Meanwhile, Hymenoptera (Apoidea) are hosts/prey of *Cacoxenus* s.str. and of some *C. (Gitonides)* species, and Coleoptera (Scolytidae) are prey of *Phortica xyleboriphaga* [12]. The substrates from which other Steganinae larvae have been bred include mostly fungi and decaying herbs or parts of logs. At least some of these may be zoophagous or zoosaprophagous; this hypothesis, proposed for *Stegana coleoptrata* 100 years ago [15], has been widely neglected. The adult stages of various Steganinae (Figures 1, 2) display a necrophilic attitude, as inferred by the relatively high attractiveness of protein traps containing dead mice to some genera of Steganinae (*Leucophenga*, *Gitona*, *Phortica*, *Amiota*) [16]. More importantly, some members of Steganinae display a clearly zoophilic tendency, feeding on mammals. For example, the adults of many species within the genera *Amiota*, *Apsiphortica* and *Phortica* may feed not only on fermenting substrates, but also on the lachrymal secretions of mammals (Figure 3). This zoophilic attitude is also referred as to lachryphagy. Since the first description of *P. variegata* imbibing the lachrymal secretions of humans, thus causing annoyance [17], many authors observed this phenomenon [18], coupled with attractiveness to animal perspiration (Additional file 1: video).

### Peculiar behaviour of lachryphagous Steganinae

Lachryphagy has been identified for many species of *Amiota* and *Phortica* - subcosmopolitan, moderately common and moderately species-rich genera, each comprising about 130 described species [10]. However lachryphagy is unknown in the *Allophortica* (subgenus of *Phortica*) comprising five described species. Flies belonging to the genera *Amiota* and *Phortica* are distributed mostly across temperate to tropical forests and lacking in arid biotopes such as those of central Asia. Conversely, the third lachryphagous genus, *Apsiphortica*, includes only six rare species in Africa and Southeast Asia. Finally, there are also single observations of lachryphagy in the genera *Gitona* [19], *Paraleucophenga* [2] and *Apenthecia* (S. Prigent, personal communication), but confirmatory studies are needed. Although the subdivision of Steganinae has not been clearly resolved, it appears that lachryphagous genera do not represent a monophyletic group [14,20]. On the other hand, the genus *Phortica*, of which adult flies display a marked lachryphagy, was recently separated from *Amiota* and its monophyly was confirmed by cladistic analyses [21–23]. Studies of the feeding habits of *Phortica variegata* confirmed previous observations, showing that whilst females prevailed on the fruit bait (male:female ratio 1:3.8), lachryphagous behaviour is exclusively by males [24]. Similar observations, although on smaller scale, exist in a number of other species of relevant genera. This behaviour is opposite to that of blood-feeding insects showing sex-related preferences (e.g., mosquitoes, ceratopogonids, sand flies, black flies and tabanids), where only adult females are haematophagous [25], with the remarkable exception of the hematophagous males of the genus *Calyptra* (Lepidoptera).

Males of *P. variegata* hover around animals and humans, possibly resting close to their eyes, imbibing tears (i.e., lachryphagy) and, occasionally, also sucking their perspiration. When trying to access their eyes, this behaviour ultimately causes a nuisance to animals [26]. Such behaviour of males is a remarkable characteristic of the abovementioned lachryphagous Steganinae (Figure 3), similarly to the members of the family Cryptochetidae (genus *Cryptochetum*), representing a sister-group of Drosophilidae [27]. Importantly, in other lachryphagous Diptera (some Muscidae, Fanniidae, Chloropidae and Paraleucopidae) females are prevalent, while the restriction of the lachryphagous behaviour to males is a rule in various Lepidoptera (except the moths *Arcyophora* and *Lobocraspis*, where both sexes are involved) [2].

Drosophilidae, and some other lachryphagous Diptera, can only feed on tears and perspiration, *Paraleucopis mexicana* can also intake blood from fresh wounds and some Chloropidae (e.g., *Liohippelates* spp.) cause direct injury except lachryphagy: they developed morphological adaptations of their mouthparts to reopen wounds of their hosts [28]. This is not the only pathway to parasitism: facultative bloodsucking moths of the genus *Calyptra* evolved from the fruit-piercing ancestors [29,30], various necrophagous flies may cause myiasis. However, lachryphagous insects feed on living animals, which makes the boundary between lachryphagy and true parasitism not obvious, and thus we tentatively consider lachryphagous behaviour as parasitical in the broad sense.

### The role of some Steganinae as vectors of helminths

Some of the lachryphagous Steganinae are known as vectors and intermediate hosts for the spirurid *Thelazia callipaeda* (Spirurida, Thelaziidae), which parasitizes the eyes of domestic and wild carnivores and some lagomorphs (see below) [31]. While feeding on animal tears, male flies have contact with the first larval stages of *T. callipaeda* and act as their intermediate hosts. In comparison to the relative species richness of lachryphagous Steganinae, strikingly few species have been confirmed as vectors of *T. callipaeda* larvae. This spirurid, known for a long time as the “oriental eyeworm” because of its occurrence in Far Eastern Countries [32], has become established in Europe [33]. Indeed, following the first description in dogs, cats and foxes in Italy [34,35] the infection has been increasingly reported in France, Switzerland, Spain, and Portugal [33] and recently in countries of the Balkans [36].

Human cases (in Europe still sparse) occur predominantly in children and elderly people and are associated with poor, rural communities and contexts of low health and socio-economic standards, where heavily infected dogs and cats live in close contact with humans [37–39]. High parasitic burdens cause various symptoms in humans such as conjunctivitis, lachrymation, corneal ulcers, rarely perforation of the cornea and even blindness [40]. In China, 84 cases of human thelaziasis were reported by the end of the 1970s and 700 additional cases between 1980 and 2006, which illustrates a rapid increase in its prevalence, although an increase in its reporting may play a role [37]. The host range of this nematode is wide as it parasitizes the eyes of dogs, cats, beech martens, foxes, wolves, rabbits, hares and humans [31]. Racoon dog (*Nyctereutes procyonoides*), a host species known from the Russian Far East [41], gained its importance as an invasive species in Europe. For a long time it was suspected that, like *Thelazia* species parasitizing cattle or horses (e.g., *Thelazia gulosa*, *Thelazia lacrimalis*, *Thelazia rhodesi*, *Thelazia skrjabini*), *T. callipaeda* has been transmitted by calyptrate flies (predominantly females) of the families Muscidae and Fanniidae to receptive animals due to their ability to suck perspiration, conjunctival liquid and exudates of their hosts. However, laboratory and field studies indicated that *Musca domestica* is not a vector of *T. callipaeda* under experimental or natural conditions [42]. The role of *Amiota nagatai* Okada, *Phortica magna* (Okada) and *P. okadai* (Máca) as vectors of *T. callipaeda* was first suggested in Japan [43,44]. In an independent study, the life cycle of *T. callipaeda* was investigated under experimental conditions in easternmost Russia [45]; the suspected vector *P. variegata* has never been confirmed later on in this area. The vectorial role of *P. variegata* (Fallén) was conclusively demonstrated under field and experimental conditions in Europe [46,47] and that of *P. okadai* (Máca) in China [48]. In addition, *P. kappa* (Máca, 1977) was found to harbour *T. callipaeda* second stage larvae [49]. Thus, the range of vectors of *T. callipaeda* is apparently limited (see above), although probably wider than the few ascertained species.

Those *Phortica* species, known to be associated with thelaziosis, belong to the subgenus *Phortica* s. str., which is widely occurring in the Palaearctic and Oriental Regions. According to the phylogenetic data on *Phortica* it can be argued that the coevolution of *T. callipaeda* with *Phortica* spp. did not begin earlier than 13.1-19.5 million years ago, when *Phortica* s. str. diverged from other clades of the genus (the time given with 95% probability) [23]. Lachryphagy, which apparently occurs more widely amongst the Steganinae, must have emerged prior to that event, or/and polyphyletically. Furthermore, the present vicariant distribution of *T. callipaeda* is likely due to parasitizing almost or completely vicariant European (*P. variegata*) and East Asian species (*P. magna*, *P. kappa*, *P. okadai*) of *Phortica* s. str. and this should be preceded by its preglacial and/or interglacial continuous Eurasian distribution. This discontinuous distribution resulted in the occurrence of a single haplotype of *T. callipaeda*, thus far, in Europe in spite of testing specimens of various European countries and as many as eight host species (e.g., dogs, cats, beech martens, foxes, wolves, rabbits, hares and humans), in contrast to the eight haplotypes found in different Far Eastern countries [50]. The low genetic variability of *T. callipaeda* in Europe seems to be in accordance with only *P. variegata* as a confirmed vector, whilst at least four species of Steganinae have been suggested to act as vectors of the eight haplotypes of this nematode found in Asian countries. This finding ultimately supports a tight affiliation of *T. callipaeda* with the ecology of the intermediate hosts.

### Distribution and host affiliation of *Thelazia callipaeda*

The distribution of *P. variegata*, the (main) European vector of *T. callipaeda*, is not known in sufficient detail for some countries [10]. Based on the ranges of temperature (i.e., 20-25°C) and relative humidity (50-75%) optimal for *Phortica* flies, as well as on the natural niche of this insect, a desktop implementation of the Genetic Algorithm for Rule-Set Prediction anticipated that large areas of Europe were likely to represent suitable habitats for *P. variegata*, therefore suggesting a potential expansion of thelaziosis [24]. Less than 10 years after this predictive niche model was published, *T. callipaeda* has been found in many areas of Europe predicted by the model [51–53]. Geographically, the prevalence of thelaziasis in different regions varies considerably in animals and humans, with most cases being recorded from the temperate to subtropical zones of the Old World, albeit in Europe only eight cases have been recorded in humans to date [33]. Case reports of human thelaziosis are much more frequent in Japan (southern part, mostly on Kyushyu island) with about 100 cases [54,55], South Korea (n = 24 cases) [55], China (about 800 cases), especially the Shandong, Hebei, Anhui and Jiansu provinces where about half of all known human cases (n = about 450) were reported [37]. The low prevalence of human thelaziosis in the Russian Far East (n = 2) may show that this region, like northern Japan, lies close to the limits of the occurrence of *T. callipaeda*, although fox farming boosted its prevalence in animals [45,56].

Interestingly, the majority of the *Phortica* s. str. species occur in the tropics of the Oriental Region, notwithstanding that data from India is sparse, most likely due to sparse use of appropriate collection methods (i.e., canopy traps). Indeed, sixty-three species of *Phortica* s. str. are known to be exclusively Oriental, six species are common to the Palaearctic and Oriental Regions, eight are exclusively Palaearctic and four are offshoots to other zoogeographical regions [57]. On the contrary, records of *T. callipaeda* from the Oriental region are sparse, with the exception of densely populated southern China, where up to 50 cases of human thelaziosis are known [37], including one case in Taiwan [58]. Cases of human thelaziosis from other countries of the Oriental Region come from India and Bangladesh (n = 10), Indonesia (n = 1), and Thailand (n = 5) [55,59,60]. The sporadic occurrence of *T. callipaeda* is well illustrated in Thailand by H. Bänziger, who anecdotally mentioned capturing 172 individuals of at least 31 species of lachryphagous drosophilids (including a few females) sucking on his eyes; in spite of that, he never mentioned any disorder of his eyes [2]. However, his collections were made in forest habitats where no or few potential hosts of *T. callipaeda* were present (Banziger, pers. comm.). As yet we do not even know the name of any of the Oriental species of Steganinae transmitting *T. callipaeda*. It should be investigated whether the Oriental species show lesser susceptibility to this nematode, or if environmental conditions of tropical humid biota represent a barrier to the perpetuation of this infection.

The host range of *Phortica* spp. is simplest in Europe, where virtually only *P. variegata* and *P. semivirgo* (and *P. erinacea* in the extreme southeast) are potential vectors of *T. callipaeda*, although just the first-mentioned species is a confirmed vector.

Vertical microdistribution (stratification) of the genera *Phortica* and *Amiota* is of epidemiological importance, considering that adult flies dwell predominantly in the tree canopies [61]. However, they fly much lower when patrolling along forest tracks and clearings, repeatedly approaching to contact objects of interest from various angles before landing on his eyes. According to H. Bänziger (personal communication) contacts with human eyes last for 35–163 seconds. Both tree canopy animals, such as beech martens, and terrestrial ones (e.g., foxes, wolves) may be contacted by these flies and infected with *T. callipaeda* larvae, whereas this may not be the case of subterranean/nocturnal European badgers [31]. Indeed, tree canopies are not the exclusive niches, at least for *Phortica* spp.; their captures in caves may indicate their facultative overwintering there [24,62,63].

### Male lachryphagy: cherchez la femme?

**Figure 4:**
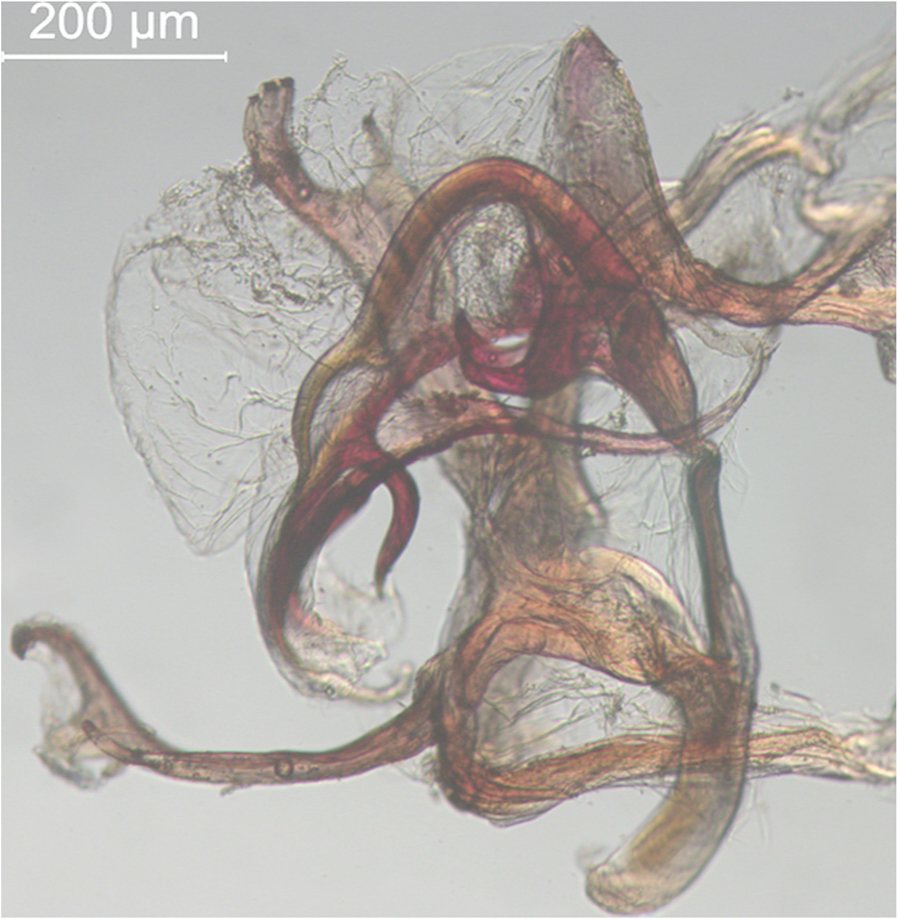
**Male genitalia of** ***Phortica variegata*** **.** Aedeagus supported by recurved rods, with extensive membranous part. Right paraphyse shown in lateral aspect, left one (lower left part of the picture) in more dorsal aspect.

**Figure 5:**
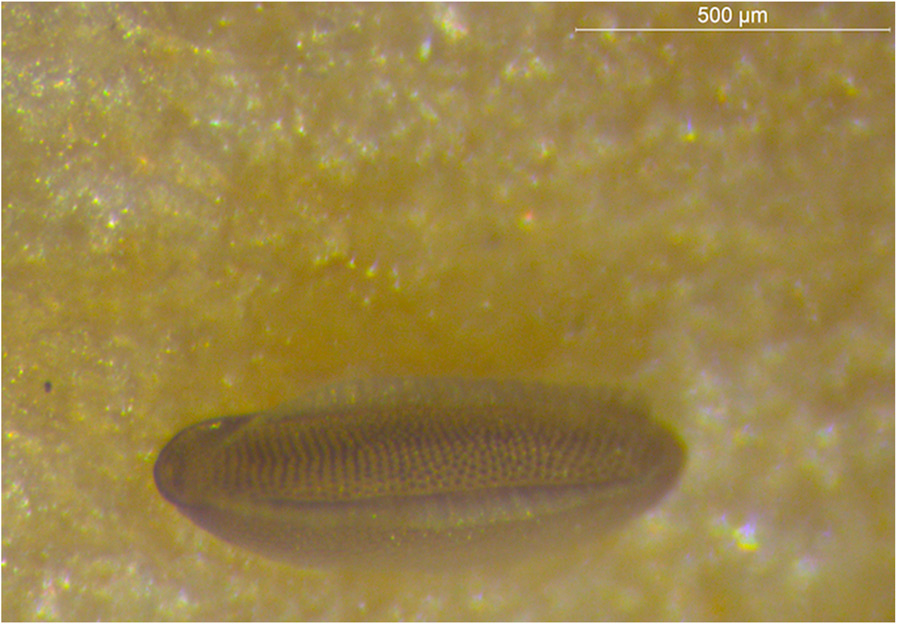
***Phortica variegata*** **,** **egg.** Like in other Steganinae, the egg possesses a pair of longitudinal vela, as opposed to Drosophilinae which presents filamentous processes. Both serve to extend the surface of the egg and enhancing oxygen supply.

**Figure 6:**
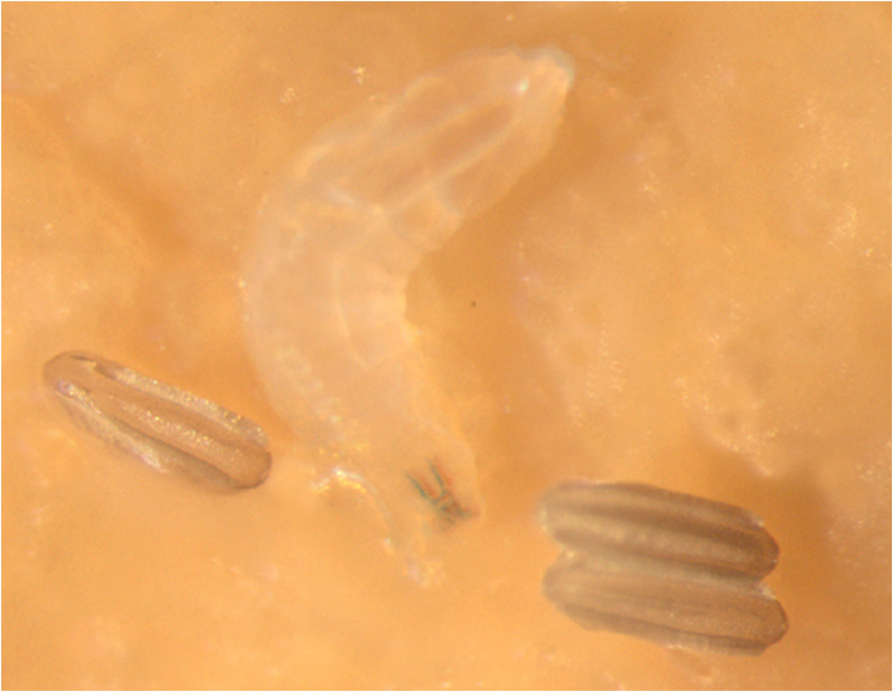
***Phortica variegata*** **,** **eggs and first instar larva.** A rare picture of the artificial breeding of *Phortica*.

Explaining lachryphagy in insects is not easy, as specific studies of this type are lacking. Lachrymal fluids contain, with the exception of salts, a certain amount of proteins, which can be utilized by the insects [64]. Protein uptake in the insect groups, where females are lachryphagous (e.g., some Muscidae and Fanniidae), may be considered parallel to the haematophagous behaviour displayed by females of various Diptera (e.g., Culicidae, Simuliidae, Tabanidae) in supporting oogenesis. However, since the lachryphagy of Drosophilidae is mostly associated with males [18], this could represent the taking of nutrients to the females as a “wedding present” [2]. This hypothesis might be supported by the fact that in spite of the rather uniform morphology of *Amiota* and *Phortica*, they present highly varied and specialized male genitalia, a recently evolved characteristic (i.e., caenogenetic structure) in contrast to most other structural characters. Indeed, in the majority of *Phortica* species, and notably in *Phortica* s. str., the aedeagus is membranous and supported by a strongly recurved medial rod, which seems to exclude direct copulation. In the related groups such as *Amiota* and *Cacoxenus* s. str., the proper aedeagus is even lacking. The ejaculatory apodeme is rudimentary to absent in *Phortica* s. str., whereas the paraphyses are as long as up to the apex of the aedeagus and apically trilobed (Figure 4). All of these characters together suggest indirect copulation (using mostly paraphyses) through a spermatophore, which could be then absorbed into the inner genitalia of the female in the same way as in *Drosophila* [65]. Therefore, following metabolism, the surplus protein taken up by the males from tears, could create the protein spermatophore needed by the female for supporting oogenesis. Another observation corroborating the hypothesis above is that the ingestion of mammalian body fluids by males precedes copulation, which allows time for metabolism of these nutrients. Indeed, females are inactive in early spring, the first generation is thus protandric and the commonly occurring aggregations of males patrolling along forest clearings have apparently no relationship with swarming [24 and our own unpublished observations]. Thus, transfer of *T. callipaeda* seems to be, after all, a by-product of the gaining of nutrients for egg development (Figure 5), although more complicated than in the case of other *Thelazia* spp., where females are lachryphagous [66]. It is apparent that studies of the mating behaviour of lachryphagous Steganinae would be most rewarding, in these regards. Unfortunately, there is no information as to whether the intake of lachrymal fluid also signals for third-stage *T. callipaeda* larvae to leave its vector, or if the presence of developing nematodes boosts the lachryphagy, or if there is no such inter-relation. Moreover, it is likely that the passage of *Thelazia* larvae to the conjunctiva of the vertebrate host may be harmful to their vector fly, considering the mechanical stress by the relative large size of the parasite and the perforation of the proboscis labellae [45; Figure 6] and possible metabolic and/or physiologic changes they may induce. This may represent the extreme sacrifice of male *Phortica* in accomplishing their mission as vectors of the “oriental eyeworm”. Still, reinfection may occur [45].

## Conclusions

Undoubtedly, various aspects of the natural history of Steganinae need to be elucidated, mostly in relation to the extent of zoophagous behavior in various genera. Refined data on this taxon of drosophilids could prove useful to the biological control of phytophagous pests from the order Homoptera, as an alternative to chemical treatment. The rare experiments carried out in this field have been referenced [12,67]. From a parasitological perspective, the reasons for the lachryphagy of adult insects remain yet to be elucidated, although the need for protein is most likely the main driver for this. Similarly, the possible role of sodium ions in this process also requires study.

Elucidation of the biology of lachryphagous drosophilids, including mating behavior, might also be addressed by rearing experiments (Figure 6). Indeed, although the rearing of *P. variegata* has been described, the results do not guarantee continual rearing [68]. Therefore, further protocols should be prepared or implemented in order to improve the rearing of Steganinae. Indeed, providing a larger space for rearing may facilitate the mating behaviour. In addition, since males are apparently protandric, and it is difficult to keep them alive whilst obtaining females, experiments with simultaneous rearing under different temperatures and light exposure may enable the study of seasonal rhythms and perhaps to align them. Although *Phortica* larvae have been found in fermenting tree sap [4], they may be virtually zoophagous as observed in *P. xyleboriphaga* [12]; fruit or other *Drosophila* breeding media do not seem suitable in supporting their development under laboratory conditions. Zoophagy in this genus is also suggested for *P*. (*Sinophthalmus*) *picta* (Coquillett) that lays either single eggs or numerous eggs for rapid propagation [69]. This egg-laying pattern might indicate parasitism and/or an adaptation for predation on gregarious prey. The possibility of larval zoophagy should be assessed by rearing *Phortica* (and allies) larvae together with those of other drosophilids (e.g. *D. melanogaster*). A better understanding of vector biology may assist not only in controlling the transmission of *T. callipaeda*, but also in discovering the origin of the predatory behaviour in this group of insects, thus leading to understanding the main drivers of their parasitism. Knowledge of the species composition of the vectors of *T. callipaeda* in Asia, mainly in its subtropical/tropical part, is still meagre. This should also be improved by implementing nematode detection in these flies as well as attempting experimental infections of various species of lacryphagous Steganinae.

## Additional file

Additional file 1:
***Phortica variegata*** **flying around human eyes.** Description: This video shows the typical questing fly of *Phortica variegata* during a summer day in Basilicata region (southern Italy), an area highly endemic for *Thelazia callipaeda*. These insects are attracted by animal perspiration and ocular secretions.
